# Carriers of human mitochondrial DNA macrohaplogroup M colonized India from southeastern Asia

**DOI:** 10.1186/s12862-016-0816-8

**Published:** 2016-11-10

**Authors:** Patricia Marrero, Khaled K. Abu-Amero, Jose M. Larruga, Vicente M. Cabrera

**Affiliations:** 1 School of Biological Sciences, University of East Anglia, Norwich, NR4 7TJ Norfolk UK; 2 Glaucoma Research Chair, Department of ophthalmology, College of Medicine, King Saud University, Riyadh, Saudi Arabia; 3 Departamento de Genética, Facultad de Biología, Universidad de La Laguna, La Laguna, Tenerife Spain

**Keywords:** Human evolution, Mitochondrial DNA, Out of Africa

## Abstract

**Background:**

From a mtDNA dominant perspective, the exit from Africa of modern humans to colonize Eurasia occurred once, around 60 kya, following a southern coastal route across Arabia and India to reach Australia short after. These pioneers carried with them the currently dominant Eurasian lineages M and N. Based also on mtDNA phylogenetic and phylogeographic grounds, some authors have proposed the coeval existence of a northern route across the Levant that brought mtDNA macrohaplogroup N to Australia. To contrast both hypothesis, here we reanalyzed the phylogeography and respective ages of mtDNA haplogroups belonging to macrohaplogroup M in different regions of Eurasia and Australasia.

**Results:**

The macrohaplogroup M has a historical implantation in West Eurasia, including the Arabian Peninsula. Founder ages of M lineages in India are significantly younger than those in East Asia, Southeast Asia and Near Oceania. Moreover, there is a significant positive correlation between the age of the M haplogroups and its longitudinal geographical distribution. These results point to a colonization of the Indian subcontinent by modern humans carrying M lineages from the east instead the west side.

**Conclusions:**

The existence of a northern route, previously proposed for the mtDNA macrohaplogroup N, is confirmed here for the macrohaplogroup M. Both mtDNA macrolineages seem to have differentiated in South East Asia from ancestral L3 lineages. Taking this genetic evidence and those reported by other disciplines we have constructed a new and more conciliatory model to explain the history of modern humans out of Africa.

**Electronic supplementary material:**

The online version of this article (doi:10.1186/s12862-016-0816-8) contains supplementary material, which is available to authorized users.

## Background

From a genetic perspective built mainly on mtDNA data, the recent African origin of modern humans [1, 2] and their spread throughout Eurasia and Oceania replacing all archaic humans dwelling there, has held a dominant position in the scientific community. The recent paleogenetic discoveries of limited introgression in the genome of non-African modern humans, of genetic material from archaic, Neanderthal [3, 4] and Denisovan [5–7] hominins has been solved adding a modest archaic assimilation note to the replacement statement [8]. In the East Asia region however, the alternative hypothesis of a continuous regional evolution of modern humans from archaic populations is supported by the slow evolution of its Paleolithic archaeological record [9] and the irrefutable presence of early and fully modern humans in China at least since 80 kya [10–12]. Moreover, recently it has been detected ancient gene flow from early modern humans into Eastern Neanderthals from the Altai Mountains in Siberia at roughly 100 kya [13]. These data contrast with the phylogenetic hypothesis of a sole and fast dispersal of modern humans out of Africa around 60 kya following a southern route [14–17]. In principle, it could be adduced, as it was in the case of the early human remains from Skhul and Qafzeh in the Levant [18], that the presence in China and Siberia of modern humans at that time was the result of a genetically unsuccessful exit from Africa. However, the fossil record shows a clinal variation along a latitudinal gradient, with decreasing ages from China to Southeast Asia [19–22] ending in Australia [23]. This gradient is in the opposite direction to the expected by the southern dispersal route. Clearly, the fossil record in East Asia would be more compatible with a model proposing an earlier exit from Africa of modern humans that arrived to China following a northern route, around 100 kya. Indeed, this northern route model was evidenced from the relative relationships obtained for worldwide human populations using classical genetic markers [24, 25] and by the archaeological record [26]. Based on the phylogeography of mtDNA macrohaplogroup N, the existence of a northern route from the Levant that colonized Asia and carried modern humans to Australia was also inferred long ago [27]. However, this idea was ignored or considered a simplistic interpretation [28]. On the contrary, since the beginning, the coastal southern route hypothesis has only received occasional criticism from the genetics field [29], and discrepancies with other disciplines were mainly based on the age of exit from Africa of modern humans [30]. However, subsequent research from the fields of genetics, archaeology and paleoanthropology [31], have given additional support to the early northern route alternative. At this respect, a recent whole-genome analysis evaluating the presence of ancient Eurasian components in Egyptians and Ethiopians pointed to Egypt and Sinai as the more likely gateway in the exodus of modern humans out of Africa [32]. Furthermore, after a thoroughly revision of the evidence in support of a northern route signaled by mtDNA macrohaplogroup N [31], we realized that the phylogeny and phylogeography of mtDNA macrohaplogroup M fit better to a northern route accompanied by N than a southern coastal route as was previously suggested [27]. In fact, M in the Arabian Peninsula seems to have a recent historical implantation as in all western Eurasia. Moreover, the founder age of M in India is younger than in eastern Asia and Near Oceania and so, southern Asia might better be perceived as a receiver more than an emissary of M lineages. Recently, the unexpected detection of M lineages in Late Pleistocene European hunter-gatherers [33] has been explained as result of a back migration from the East, possibly mirroring the arrival to Africa of the haplogroup M1 in Paleolithic times [34–36], although a more ambitious interpretation has been formulated by others [37]. In this study, we propose a more conciliatory model to explain the history of Homo sapiens in Eurasia under the premise of an early exit from Africa following a sole northern route across the Levant to colonize the Old World.

## Methods

### Sampling information

A total of 206 samples from unrelated Saudi healthy donors belonging to mtDNA macrohaplogroup M were analyzed in this study, 163 of them previously published [31, 38]. To fully characterize these M lineages, 17 complete mtDNA genomes from Saudi samples were sequenced. In addition, 7 unpublished complete mtDNA genomes from preceding studies were included [35, 39]. Only individuals with known maternal ancestors for at least three generations were considered in this study. Moreover, 4107 published complete or nearly complete mtDNA genomes belonging to macrohaplogroup M of Eurasian and Oceania origin were included in the analysis. To accurately establish the geographical ranges of the relatively rare M haplogroups, 73,215 partial sequences from the literature were screened as detailed in [31]. The procedure of human population sampling adhered to the tenets of the Declaration of Helsinki and written consent was recorded from all participants prior to taking part in the study. The study underwent formal review and was approved by the College of Medicine Ethical Committee of the King Saud University (proposal N° 09-659) and by the Ethics Committee for Human Research at the University of La Laguna (proposal NR157).

### MtDNA sequencing

Total DNA was isolated from buccal or blood samples using the POREGENE DNA isolation kit from Gentra Systems (Minneapolis, USA). The I and II hypervariable regions of mtDNA from 43 new Saudi Arabian samples were amplified and sequenced for both complementary strands as detailed in [40]. When necessary for unequivocal assortment into specific M subclades, the 206 Saudi M samples were additionally analyzed for haplogroup diagnostic SNPs using partial sequencing of the mtDNA fragments including those SNPs, or typed by SNaPshot multiplex reactions [41]. PCR conditions and sequencing of mtDNA genome were as previously published [27]. Successfully amplified products were sequenced for both complementary strands using the DYEnamic™ETDye terminator kit (Amersham Biosciences). Samples run on MegaBACE™ 1000 (Amersham Biosciences) according to the manufacturer’s protocol. The 24 new complete mtDNA sequences have been deposited in GenBank with the accession numbers KR074233 to KR074256 (Additional file 1: Figure S1).

### Previous published data compilation

Sequences belonging to specific M haplogroups were obtained from public databases such as NCBI, MITOMAP, the-1000 Genomes Project and the literature. We searched for mtDNA lineages directly using diagnostic SNPs (http://www.mitomap.org/foswiki/bin/view/MITOMAP/WebHome), or by submitting short fragments including those diagnostic SNPs to a BLAST search (http://blast.st-va.ncbi.nlm.nih.gov/Blast.cgi). Haplotypes extracted from the literature were transformed into sequences using the HaploSearch program [42]. Sequences were manually aligned and compared to the rCRS [43] with BioEdit Sequence Alignment program [44]. Haplogroup assignment was performed by hand, screening for diagnostic positions or motifs at both hypervariable and coding regions whenever possible. Sequence alignment and haplogroup assignment was carried out twice by two independent researchers and any discrepancy resolved according to the PhyloTree database [45].

### Phylogenetic analysis

Phylogenetic trees were constructed by means of the Network v4.6.1.2 program using the Reduced Median and the Median Joining algorithms in sequent order [46]. Resting reticulations were manually resolved attending to the relative mutation rate of the positions involved. Haplogroup branches were named following the nomenclature suggested by the PhyloTree database [45]. Coalescence ages were estimated by the statistics rho [47] and sigma [48], and the calibration rate proposed in [49]. Differences in coalescence ages were calculated by two-tailed t-tests. It was considered that the mean and standard error estimated for haplogroup ages from different samples and methods were normally distributed.

### Global phylogeographic analysis

In this study, we deal with the earliest periods of the out of Africa spread. Given that subsequent demographic events probably eroded those early movements, spatial geographical distributions of haplogroups based on their contemporary frequencies or diversities were omitted. The presence/absence of basal M lineages in different areas was used to establish the present day geographical range for each haplogroup. Haplogroups with extensive geographic overlapping were considered as belonging to the same sub-continental region. AMOVA, CLUSTER and PCA analysis were performed to evaluate the level of geographical structure of the M haplogroups. For AMOVA we used the GenAlEx6.5 software, k-means clustering was obtained with XLSTAT statistical software, and PCA was performed using the Excel add-in Multibase package (Numerical Dynamics, Japan). The possible association between the haplogroup and fossil ages with their respective longitudinal and latitudinal geographical positions was tested by Pearson correlation analyses using the XLSTAT statistical software. For this purpose we took the intersection point between the two segments joining the largest latitudinal and longitudinal areas of each haplogroup as its geographic center. Geographic coordinates were obtained by Google Earth software (https://earth.google.com).

### Geographic subdivision of India and regional haplogroup assignation

Attending only to geographic criteria, India was roughly subdivided into four different sampling areas: Northwest, including Kashmir, Himachal Pradesh, Punjab, Haryana, Uttarakhand, Rajasthan, Uttar Pradesh, Gujarat, and Madhya Pradesh states; Southwest, including Maharashtra, Karnataka and Kerala; Northeast, including Bihar, Sikkim, Arunachal Pradesh, Assam, Nagaland, Meghalaya, Tripura, Jharkhand, West Bengal, Chhattisgarh and Orissa; and Southeast, represented by Andhra Pradesh, Tamil Nadu and Sri Lanka. We are very skeptical of the possibility that the actual genetic structure of India is the result of its original colonization, so the ethnic or linguistic affiliation of the samples were not considered but only its geographic origin. For the same reason, present day frequency and diversity of the haplogroups were not used but their geographic ranges and radiation ages. The criteria followed to assign haplogroups to different regions within India were a consistent detection in an area (at least 90 % of the samples) and absence or limited presence in the alternative areas (equal or less than 10 % of the samples). We considered widespread those haplogroups consistently found in all the Indian areas and also found in some of the surrounding areas as Pakistan or Iran at the west, Tibet or Nepal at the north, and Bangladesh or Myanmar at the east.

## Results and discussion

### Haplogroup M in western Eurasia with emphasis on Saudi Arabia

The lack of ancient and autochthonous mtDNA M lineages in western Eurasia is, at least, surprising if the colonization of the Old World by modern humans is thought to have begun through that region. Indeed, the presence of haplogroup M1 lineages in Mediterranean Europe and the Middle East has been explained as the result of secondary spreads from northern Africa where this haplogroup had a Paleolithic implantation [34–36]. Likewise, eastern Asian M lineages belonging to C, D, G and Z haplogroups mainly in Finno-Ugric-speaking populations of north and eastern Europe, seem to be the footprints of successive westward migration waves of Asiatic nomads occurred from Mesolithic period to historic times [50–58]. South Asian influences on the west have been also evidenced by the presence of Indian M4, M49 and M61 lineages in Mesopotamian remains [59]. In addition, ancestral mtDNA links between European Romani groups and northwest India populations were proved by the sharing of M5a1, M18, M25 and M35b lineages [60–64].

The mtDNA haplogroup M profile in Saudi Arabia represents about 7 % of the maternal gene pool [38, 65]. Of the 206 M haplotypes sampled (Additional file 2: Table S1), 53 % belong to the northern African M1 haplogroup, being both eastern African M1a and northern African M1b branches well represented (Additional file 2: Table S1 and Additional file 1: Figure S1). This fact contrasts with the sole presence of eastern M1a representatives in Yemen [66]. Therefore, it is most probably that M1b lineages reached Saudi Arabia from the Levant. M lineages with indubitable Indian origin accounted for 39 % of the Saudi M pool, whereas the resting 8 % would have a southeastern Asian source. The geographic origin of the Indian contribution seems not to be biased since 53 % of the lineages may be assigned to eastern Indian regions and 47 % to western [67–69]. However, it deserves mention that the two M lineages of the Andaman aborigines [15, 70, 71] are present in the Saudi samples. The isolate Ar2461 (Additional file 2: Table S1) has the diagnostic mutations of the Andaman branch M31a1 in the regulatory (249d, 16311) and coding (3975, 3999) regions. On the other hand, the complete mtDNA sequence of Ar1076 (Additional file 1: Figure S1) belongs to the M32c Andamanese branch, matching it with another complete sequence from Madagascar [72]. It must be stressed that this branch has been steadily found in all mtDNA reports on Madagascar [73–75]. Although these haplogroups were taken as evidence that Andamanese indigenous represent the descendants of the first out of Africa dispersal of modern humans [15, 70], more recent studies support a late Paleolithic colonization of the Andaman Islands [76, 77]. In fact, different branches of M31 have been found in the northeastern India, Nepal and Myanmar [71, 77–80], while M32c has been also detected in Indonesia [81] and Malaysia [82, 83]. Another interesting link involving India, Saudi Arabia and the Mauritius Island is the case of the haplogroup M81. It was first detected as a sole sequence in a LHON patient from India [84]. The Saudi Ar567 sample shares 215, 4254, 6620, 13590, 16129 and 16311 substitutions with this Indian sequence and, in addition it shares substitutions 151, 6170, 7954 and 16263 with a complete sequence from the Mauritius Island [85]. At first sight, the Mauritius sequence differs from that of Saudi Arabia by four different mutations occurring in a short segment of 11 bp, from 5742 to 5752. However, a closer inspection reveals they may represent two different interpretations: the C to G transversion at position 5743 in the Ma12 reading corresponds to the C deletion at the same position in the Ar567 lecture, and the G to A transition at the position 5746 in Ma12 corresponds to the A insertion at the position 5752 in Ar567. Therefore, both sequences can be only distinguished by the transition 12522, present in the Saudi sample and absent in Ma12 (Additional file 1: Figure S1). Curiously, these affinities between samples from Saudi Arabia and those from Indian Ocean islands can be extended to Saudi Q1a1 in Ar196 (Additional file 2: Table S1), which has only exact matches with MA405 sample from Madagascar [75]. Moreover, different lineages belonging to the Indian branch (M42b) in Saudi Arabia [31] and Mauritius [85] deeply link with the Australian M42a branch [86]. Other lineages in the Saudi mtDNA pool, such as M20, E1a1a1 and M7c1 point to specific arrivals from southeastern Asia. The fact that all these lineages represent isolates in Saudi Arabia closely related to those found in their original areas, strongly supports the idea that they were incorporated to the Saudi pool in historical times as result of the Islamic expansion and by the import of workers from India and southeastern Asia by the European colonizers in the past and by the own Saudi nowadays [38]. In conclusion, Saudi Arabia lacks of ancient and autochthonous M haplogroups likewise the rest of western Eurasia.

### About the origin of the North African haplogroup M1

The existence of haplogroup M lineages in Africa was first detected in Ethiopian populations by RFLP analysis [87]. Although an Asian influence was contemplated to explain the presence of this M component on the maternal Ethiopian pool, the dearth of M lineages in the Levant and its abundance in south Asia gave strength to the hypothesis that haplogroup M1 in Ethiopia was a genetic indicator of the southern route out of Africa. In addition, it was pointed out that probably this was the only successful early dispersal [88]. However, the limited geographic range and genetic diversity of M in Africa compared to India was used as an argument against this hypothesis [27, 34, 35, 67, 78, 89], instead proposing M1 as a signal of backflow to Africa from the Indian subcontinent. However, after extensive phylogenetic and phylogeographic analyses for this marker [34–36, 67, 90], the supposed India to Africa connection was not found.

The detection in southeast Asia of new lineages that share with M1 the 14110 substitution [90, 91], gave rise to the definition of a new macrohaplogroup named M1′20′51 by PhyloTree.org Build 16 [44]. However, this substitution is not an invariable position (Additional file 2: Table S2) and, therefore, its sharing by common ancestry is not warranted [36]. We realized that, in addition, haplogroups M1 and M20 share transition 16129 which, although highly variable, would add support to this basal unification as the most parsimonious phylogenetic reconstruction (Additional file 1: Figure S1). However, a recent study reported a new mtDNA haplogroup from Myanmar, named M84, that also roots at the basal node represented by transition 14110 [92]. This haplogroup shares with M20 the transition 16272 which is more conservative than 16129 [48], therefore, weakening any specific relationship between haplogroups M1 and M20 beyond its common basal node. With the exception of M84, that seems to be limited to Myanmar, India and Southern China populations [92], the phylogeography of these haplogroups is extend and complex (Additional file 2: Table S3). M1 is found from Portugal and Senegal in the west to the Caucasus, Pakistan and Tibet at the east and, from Guinea-Bissau and Tanzania in the south to Russia at the north [34–36, 93–98] but, its highest diversity is found in Ethiopia and the Maghreb. The isolates detected at the borders are lineages derived from the M1a branch in Russia and Tanzania and the M1b branch in Guinea Bissau and Tibet. The geographic ranges of M20 and M51 largely overlap showing a broad common area in the southeastern Asia including Myanmar and Malaysia at the west, Philippines and Hainan at the east and Tibet at the northwest (Additional file 2: Table S3). In addition, the western border of M20 further stretches to Bangladesh [99] and even to Assam in India [100] and the eastern border of M51 to Fujian and Taiwan [91]. The primary split between the ancestors of these four haplogroups probably occurred in its core area around 56 kya, coinciding with a warm climate period. Thus, it might be possible that the birth of the M1 ancestor was in southeastern Asia instead of India. Based on the scarcity and low diversity of M1 along the southern route [66], and on archaeological affinities between Levant and North Africa [34, 35], it has been suggested that the route followed by the M1 bearers to reach Africa was across the Levant not to the Bab el Mandeb strait. However, the current representatives of M1, from the Levant to the Tibet, are derived lineages of the ancestors in Africa. Until now, basal lineages of M1 have not been detected in the northern and southern hypothetical paths, so the only support for a Levantine route of M1 is the fact that all the other Eurasian lineages that returned to Africa in secondary backflows, such as N1, X1 or U6, had their most probable origins in the central or western Eurasia instead of India.

### Geographical structure of the macrohaplogroup M genealogy

**Fig. 1:**
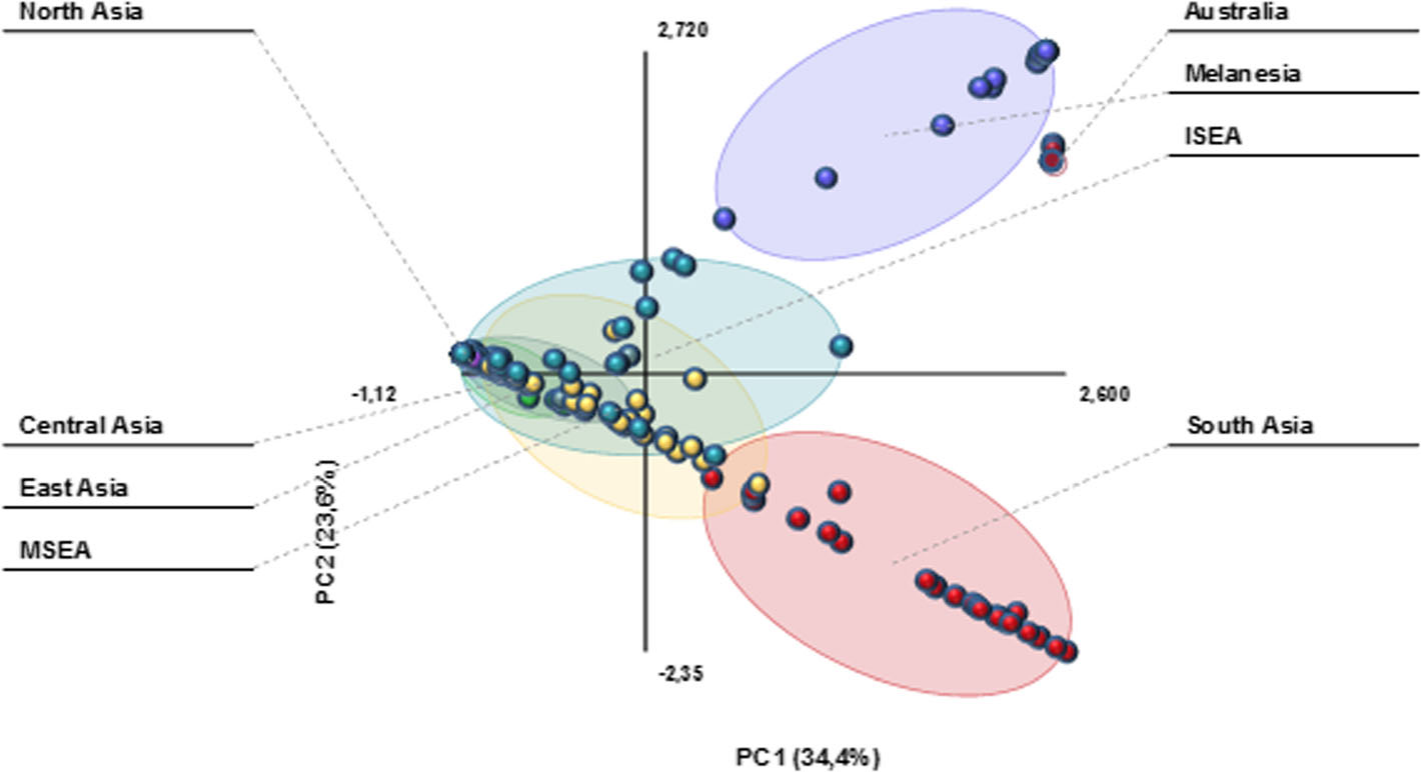
PCA plot showing the level of geographical structure of the M haplogroups

At global level, the mtDNA variation is phylogeographically structured [101]. For macrohaplogroup M, the regions of South Asia, East Asia and Southeast Asia have their characteristic sets of haplogroups with only minor overlapping. The same occurs in Melanesia and Australia. It is of paramount importance point out that these sets of haplogroups only share diagnostic mutations defining the basic M* node. This picture is interpreted as the result of secondary expansions from several geographically isolated centers which were reached by carriers of basic M* lineages during the primary earlier migrations. Congruently, the AMOVA analysis of 176 populations covering the main regions of Asia, Melanesia and Australia (Additional file 2: Table S4), shows that 85 % of the variance was found within populations and 15 % among the major regions (*p* < 0.0001). Furthermore, when populations were successively partitioned into k-clusters in order to minimize the within-cluster variance, the best partition was obtained for k = 5 (Table 1). The major regional differences explained 90.55 % of the variance. At this level, three clusters grouped together populations only belonging to Australia, Melanesia and South Asia respectively, a fourth cluster joined all Central Asian populations and the majority of the East and North Asian populations together with a few Mainland (4) and Island (8) southeast Asian populations. Finally, the fifth cluster comprised the majority of the Mainland and Island southeast Asian populations and a few East (2) and South (3) Asian populations. These results are graphically visualized in the PCA plot (Fig. 1) where the first (34.4 %) and second (23.6 %) components accounted for 58 % of the variability. South Asia, Melanesia and Australia are nearly disjoint areas whereas the rest show important overlapping. As these regional genealogies can be transformed in coalescence ages, the relative role of each sub-continental area in the primitive human migrations can be approached.

**Table 1:** AMOVA and k-mean Clustering results

Statistic	Variance (%)	
	Within populations	Between regions
AMOVA	85.00	15.00
k-2 Clustering	54.27	45.73
k-3 Clustering	33.00	67.00
k-4 Clustering	21.94	78.06
k-5 Clustering	9.45	90.55

### The role of India in the origin and expansion of the macrohaplogroup M

The macrohaplogroup M in South Asia is characterized by a great diversity, deep coalescence age, and autochthonous nature of its lineages. These characteristics have been used for support the in-situ origin of the Indian M lineages and its rapid dispersal eastwards to colonize southeastern Asia following a southern coastal route [14, 15, 68]. However, there are several arguments against this hypothesis. Firstly, given that geographical barriers seem not to be stronger at the western border than at the eastern border of the subcontinent [102], it should be expected that the macrohaplogroup M would simultaneously radiated to both areas with India as its first center of expansion. However, while at the east its expansion crossed the Ganges and quickly reached Australia, at the west it seems to have found an insurmountable barrier at the Indus bank since M frequencies suddenly dropped from 65.5 ± 4.3 % in India to 5.3 ± 1.0 % in Iran (*p* < 0.0001) [67, 103–105]. Furthermore, unlike southeastern Asia and Australia, autochthonous M lineages have not been detected at the west of South Asia. It might be adduced that later massive expansions of western mtDNA linages replaced the M lineages in those regions, but these western linages have not been detected in India. Instead, it has been pointed out that the most of the extant mtDNA boundaries in South and southwest Asia were likely shaped during the initial settlement of Eurasia by anatomically modern humans [67]. In fact, only a small fraction of the specific western Asia mtDNA lineages found in Indian populations can be ascribed to a relatively recent admixture [106].

Secondly, whenever it has been compared, the founder age of M in India is significantly (*p* = 0.002) younger than those in eastern and southeastern Asia and Australo-melanesian centers (Table 2). It has been argued that this could be due to an uneven distribution and sampling of M lineages in India [78, 90]. However, when the same weight is given to every lineage independently of its respective abundance (Table 1), the founder age slightly diminish in India and raises in East Asia compared to their respective weighted founder ages. Recently, it has been admitted that the initial radiation of macrohaplogroup M could have occurred in eastern India following the southern route [17]. In fact, the possibility that the first radiation of M were in East Asia instead of India was proposed long ago [27]. We think that it would be a hard task to detect today the original focus of the macrohaplogroup M expansion into India if it really occurred. Indeed, the mean radiation age (30.1 ± 9.3 ky) of the M haplogroups, that could be considered of widespread implantation in India, was significantly older (Two-tailed p value = 0.0147) than the mean radiation age (22.2 ± 9.6 ky) of those with more localized ranges (Additional file 2: Table S3). However, comparisons between mean radiation ages of Indian M haplogroups with eastern vs western (*p* = 0.275) and northern vs southern (*p* = 0.580) geographic ranges were not statistically significant. Furthermore, it must be taken into account the possibility that some M haplogroups with dominant implantation in northeastern India, such as M49, are secondary radiations from Myanmar [107, 108]. It is evident that the founder age of macrohaplogroup M increases eastwards from South Asia. Not only is the founder age of M in East Asia significantly greater than in South Asia but, the former is younger than the one estimated for southeast Asia despite of the probability value (*p* = 0.0998) did not reach significance probably because the estimation for the area calculated by [90] was previous to the detection of M clades with very deep ages in southeast Asia [91, 92, 109]. In turn, the founder age for M in Oceania was significantly older than the ones estimated for East Asia (*p* = 0.004) and southeast Asia (*p* = 0.032). Furthermore, there is a significant positive correlation (*R* = 0.554; *p* < 0.0001) between the age of the M haplogroups and its relative longitudinal position (Additional file 2: Table S4). This decreasing age gradient westwards is in accordance with the hypothesis that carriers of macrohaplogroup M lineages colonized India from the East instead the West. It could be argued that South Asia was eastward colonized very early by the southern coastal expansion of modern humans out of Africa but those primitive mtDNA lineages were extinguished by genetic drift, and the subcontinent was recolonized latter by eastern groups left on the way to Australia. This argument is however in contradiction, first, with the suggestion based on past population size prediction, that the most of humanity lived in southern Asia approximately between 45 and 20 kya [110], and second, with the significantly older (*p* = 0.004) mean founder age of macrohaplogroup R in South Asia (62.5 ± 3.5 ky) than its M counterpart (45.7 ± 7.8 ky). These data are in agreement with a colonization of South Asia by two independent waves of settlers, already proposed for Indian populations according to the phylogeny and phylogeography of M and U haplogroups [106]. Curiously, this idea was abandoned in favor of a single southern route simultaneously carrying the three Eurasian mtDNA lineages (M, N and R). Now, if the born of macrohaplogroup M at some place between southeast Asia and near Oceania is accepted, the putative connection between Australia and India based on the nearly simultaneous radiation of M42a and M42b branches respectively [86] could be explained by the expansion from a nearly equidistant center of radiation, and not as a directional colonization from India to Australia following a southern route. At this respect, it seems pertinent to cite that the complexity of M42 in Australia could be greater than the currently known [111]. In addition, the tentative junction of M42 with the specific southeast Asian M74 lineage [45] based on a shared transition at 8251 gives additional support to this hypothesis.

**Table 2:** Founder ages (kya) for macrohaplogroup M in South and East Asia

South Asia	East Asia	SoutheastAsia	Oceania^a^	References
40.2 (20.8; 60.9)	55.2 (26.0; 86.7)			This study
38.4 (19.1; 58.9)^b^	56.4 (27.1; 88.1)^b^			This study
	51.6 (40.4; 63.1)	60.7 (47.4; 74.4)	68.3 (53.5; 83.6)	This study
			72.1 ± 8.0	[142]
44.6 ± 3.3	69.3 ± 5.4	55.7 ± 7.4	73.0 ± 7.9	[89]
		58.9 ± 13.6		[80]
66.0 ± 9.0^c^	69.0 ± 7.0^c^			[67]
36.0 ± 3.0^d^	46.0 ± 5.0^d^			[67]
49.4 (39.0; 60.2)	60.6 (47.3; 74.3)			[48]

^a^Including Australia as in [143]

^b^Unweighted rho

^c^Based on coding region only

^d^Based on synonymous positions only

### Basal M superhaplogroups involving South Asia

As the most parsimonious topology, the existence of several M superhaplogroups, embracing different lineages based on one or a few moderately recurrent mutations have been recognized in PhyloTree.org Build 16 [44]. Following this trend we have, provisionally, unified haplogroup M11 and the recently defined haplogroup M82 [92] under macrohaplogroup M11′82 because both share the very conservative transition 8108 (Additional file 2: Table S2). Attending to their phylogeography these superhaplogroups usually link very wide geographic areas (Additional file 2: Table S2). In accordance with our suggestion that the carriers of Macrohaplogroup M colonized South Asia later than southeastern Asia and Oceania and that this mtDNA gene flow had an eastern origin we have observed that the mean radiation age of those superhaplogroups involving South Asia (39.70 ± 3.24 ky) is significantly younger (*p* = 0.003) than the one relating East and Southeast Asia (55.60 ± 2.94 ky). In this comparison we considered the South Asia-Southeast Asia-Australian link deduced from superhaplogroup M42′74 as specifically involving India. However, we considered superhaplogroup M62′68 as indicative of an East Asia-Southeast Asia link because M62 has been found consistently in Tibet [112, 113] but only sporadically in northeast India [67].

### The perspective from other genetic markers

Based on the absence of autochthonous N lineages in India and their deep age in southeastern Asia, we recently reasserted [31] the hypothesis that mtDNA macrohaplogroup N reached Australia following a northern route [27]. This time, we found additional support from Paleogenetics, which has demonstrated the introgression of DNA from Neanderthals [3, 4] and Denisovans [7, 114] of most probably northern geographical ranges, into the genome of modern humans [115]. For the specific case of India, we proposed that the N and R lineages arrived as secondary waves from the north following the Indus and Ganges-Brahmaputra banks. In our opinion, India was first colonized by modern humans from two external geographical centers situated at the northwest and northeast borders of this subcontinent [31]. The same picture was depicted, long ago, by other authors also based on mtDNA variation in India [106], although suggesting an eastward southern route for macrohaplogroup M in the same way as proposed by us [27]. Due to the lack of any mtDNA genetic evidence in support of an early migration along the southern route, we now propose an early exit of modern humans from Africa by the Levant and a unique northern route up to the Altai Mountains and then, obliged by harsh weather, down to southern China and beyond [31]. Strong evidence, supporting the primitive colonization of India by modern humans in two waves, has come from wide genome analysis. After analyzing 132 samples from 15 Indian states for more than 500,000 autosomal SNPs, the existence of two ancient populations genetically divergent was detected in India [116]. These are the ancestral northern Indians close to middle Eastern, central Asians and Europeans, and the ancestral southern Indians, distinct from the northern component and East Asians as they are from each other. Actually, the southern component is the most prevalent population in Andaman. Of particular interest is the fact that when populations of southeast Asia and Near Oceania were incorporated to these broad genome analyses, the Andaman Islanders showed a closer affinity with southeast rather than South Asian populations [117, 118]. It is evident that our mtDNA interpretation and the autosomal results give a very similar picture. In addition, it has been documented recently more Denisovan ancestry in South Asia than is expected based on existing models of history [119] but, again, the decreasing gradient of Denisovan ancestry detected from Oceanian populations to southeast and southern Asian populations easily fits in our model of an early expansion of macrohaplogroup M from the East to colonize the Indian subcontinent. Furthermore, independent support for the existence of an early center of primitive modern humans in southeastern Asia originating very early expansions has recently come from improved phylogenetic resolution of the Y-chromosome K-M526 haplogroup. It has been detected a rapid diversification process of this haplogroup in southeast Asia-Oceania with subsequent westward expansions of the ancestors of R and Q haplogroups that make up the majority of paternal lineages in central Asia and Europe [120].

### The fossil evidence

There is strong contradiction between the paleontological and the genetic interpretations about the origin of modern human in East Asia. New and reliable chronometric dating techniques applied to morphologically classified human remains have demonstrated the presence of early and fully modern humans in southern China at least since 80 kya which is in accordance to a regional continuity, or in situ evolution, of modern humans in East Asia [11, 121]. On the other hand, no apparent genetic contribution from earlier hominids was detected in the maternal [39, 91] and paternal [122–124] genetic pools of extant East Asian populations which has been taken as in support of a recent replacement of archaic humans by modern African incomers in East Asia. Although recently, genome analysis have detected introgression of Neanderthal [4] and Denisovan [7] DNA to the extant [125] and the ancient [126] genomes of modern humans in East Asia, this genetic contribution can be explained as a limited assimilation episode. Ancient DNA analysis of a morphologically early modern human from Tianyuan cave in northeast China [126] and a modern human from western Siberia of 45 ky old [127] evidenced that both were bearers of B and U mtDNA lineages respectively. These lineages are branches of haplogroup R which, in its turn, derives from macrohaplogroup N indicating thus that people in North Asia carried already fully modern mtDNA lineages around 45 kya, which is a hint of a northward secondary expansion of modern humans at that time. It seems pertinent to mention here, that in this study we found a weak but significant negative correlation (*r* = -0.254; *p* = 0.029) between the age of M haplogroups and their latitudinal geographical centers. On the other hand, dates of the East Asian fossil record (Additional file 2: Table S4) also showed a significant positive correlation (*r* = 0.772; *p* = 0.0008) with their respective southward latitudes from China. This is in agreement with our proposition that the out of Africa of modern humans occurred across the Levant and that the Skhul and Qafzeh fossil remains in Israel could be the first landmarks of that successful exit [31]. The subsequent evolution of those early modern humans in Asia might reconcile the replacement and continuity models into an inclusive synthesis. On the mtDNA side, the main problem for this reconciliation would be an strict adhesion to the chronological upper bound marked for the African exit by the coalescent age of macrohaplogroup L3 [17]. However, we think that mtDNA dating methods are still not reliable in absolute terms, mainly because we need accurate independent calibration for deep nodes and, in addition to selection, to take into account the effects of demographic parameters on the temporal variation of the substitution rate.

Turning to the probable existence of a primitive center of modern human expansion in southeast Asia, as proposed here on the basis of mtDNA haplogroup M phylogeography, the existence of a positive westward longitudinal gradient (*r* = 0.551) of the fossil record dates in southeastern Asia, with the older ages in Philippines and the youngest in Sri-Lanka (Additional file 2: Table S4), deserves mention. However, the correlation this time was not statistically significant (*p* = 0.199), mainly due to the lack of Paleolithic fossil evidence in Myanmar and India. Certainly, this counter-clock trend has been perceived by other authors as an argument against the recent straight forward out of Africa dispersal of Homo sapiens model [102].

### The archaeological evidence

The archaeological record in East Asia also seems to be in support of the regional continuity model. The persistence and dominance of simple core-flake assemblages throughout the Paleolithic in this area sharply contrasts with the Upper Paleolithic technical and cultural innovations in western Eurasia [128]. Different lithic assemblages with potential resemblances in Africa and in southeastern Asia are crucial to interpret the southern dispersal route of modern humans across India. Blade-microblade and core-flake technologies are both present in the Indian subcontinent. They are usually contemporary and, in some cases, core-flake industries, such as the Soanian, even post-date the former [129]. Microlithics have been dated around 35-30 kya in southern India and Sri Lanka [130], although recently, it has been established that the microblade technology presents continuity in central India since 45 kya [131]. Some authors have proposed that the Indian microlithics had an African original source [17]. This model has problems to explain the absence of microblades eastwards of the subcontinent around that time, and the chronological gap between the oldest microlithic dates in India and the arrival of modern humans to Australia. Other authors consider microlithics in India as local innovations [130], however, the absence of an earlier blade technology in the Indian Paleolithic record makes an indigenous development unlikely [131]. Finally, from the age of the microblade technology in India, other authors have deduced that modern humans skirted the Indian subcontinent in the first dispersal out of Africa taking a northerly route through the middle East, central Asia, and southeastern Asia across southern China [131]. For these authors, modern humans did not actually enter India until the time marked by the glacial climate of MIS 4. On the other hand, some core and flake industries in India have been considered as a link between those present in sub-Saharan Africa, southeast Asia and Australia [132–134]. Core sites in India with ages around 77 ky would be compatible with an early dispersal of modern humans from Africa. This model confront problems with the later mtDNA molecular clock boundary proposed by some geneticists [16] and the lack of contemporary fossil record to confirm that this primitive technology was manufactured by modern humans. Finally, some Indian Late Pleistocene core-flake types as the Soanian seem to be related to similar industries in southeastern Asia, such as those of the Hoabinhian [135]. We suggest that microlithic technology could show the arrival of mtDNA macrohaplogroup R to India, following the northwest passage, as a branch of a global secondary dispersal of modern humans from some core area in west/central Asia that also affected Europe [26] and that later, reached North China across Siberia and Mongolia [136]. On the other hand, macrohaplogroup M came to India from southeast Asia following the northeast passage and carrying with them a simple core-flake technology.

## Conclusions

**Fig. 2:**
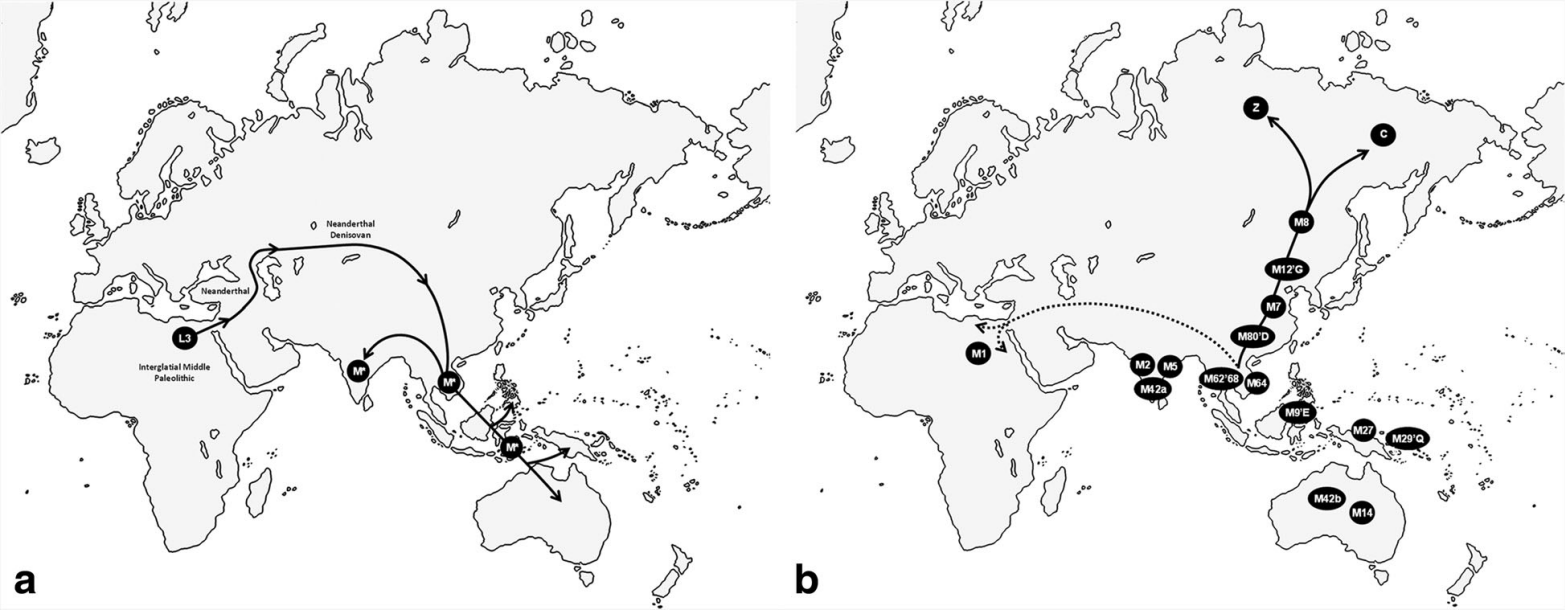
Proposed routes followed by modern humans in their exit from Africa: (**a**) Northern route to reach South Asia, the Philippines and nearly Oceania, and (**b**) secondary expansions northward through Asia to the Americas and southwest to North Africa and Europe

A new and integrative model, explaining the time and routes followed by modern humans in their exit from Africa, is proposed in this study (Fig. 2). First, we think that the exit from Africa followed a northern route across the Levant, and that the fossils of early modern humans at Skhul and Qafzeh could be signals of this successful dispersal. These first modern humans carried undifferentiated mtDNA L3 lineages and brought primitive core-flake technology to Eurasia [26]. The dates estimated for Skhul and Qafzeh remains (Additional file 2: Table S4) are slightly out of the range calculated for the age of mtDNA macrohaplogroup L3 (78.3, 95 % CI: 62.4; 94.9 kya) based on ancient mtDNA genomes [137]. However, they are in accordance with the presence of early modern humans in China around 100 kya, and with their subsequent presence in southeastern Asia about 70 kya. If we add the fact, also based on ancient genomes, that mtDNA lineages in northern Asia already belonged to derived B and U haplogroups around 45 kya [126, 127], we opine that the geneticists should resynchronize the mtDNA molecular clock with the Levant and East Asia fossil records instead of consider them as result of unsuccessful migrations. Second, those early modern humans went further northwards, some at least to the Altai Mountains, and in the way they occasionally mixed with other hominids as Neanderthal and Denisovans. Harsh climatic conditions dispersed them southwards erasing the mtDNA genetic footprints of this pioneer northern phase [31]. Third, the small surviving groups already carried basic N and M lineages. One of them, with only maternal N lineages, spread southwards to present-day southern China and probably, across the Sunda shelf reached Australia and the Philippines [31]. Fourth, other dispersed groups were the bearers of other N branches, including macrohaplogroup R, that enter India from the north, carrying with them the blade-microblade technology detected in this subcontinent. This technology also spread with other N and R branches to northern and western Eurasia, reaching Europe, the Levant and even northern Africa. Fifth, short after, another southeastern group carrying undifferentiated M lineages radiated from a core area, most probably localized in southeast Asia (including southern China), reaching India westwards and near Oceania eastwards. These M haplogroup bearers brought with them at least one of the primitive core-flake technologies present in India that, therefore, had to have a southeastern Asian origin. Sixth, in subsequent mild climatic windows, demographic growth dispersed macrohaplogroup M and N northwards, most probably from overlapping areas that in time colonized northern Asia and the New World.

It seems that under archaeological grounds there are data in support of a southern route exit from Africa and across the Arabian Peninsula and the Indian subcontinent [30, 130, 132, 133, 138–141], but the lack of coetaneous fossil record leaves the hominid association to these stone tools unresolved. Even if they were modern humans, they did not leave any trace in the mtDNA gene pool of the extant populations of Arabia or India. However, there is no necessity to invoke the existence of a southern route to interpret the landscape depicted by the mtDNA phylogeny and phylogeography.

## Additional files

Additional file 1: Figure S1. Phylogenetic tree of M complete sequences from this study. (XLSX 66 kb)
Additional file 2: Table S1. Haplogroup M in Saudi Arabia. In bold haplotypes from this study. Table S2. Super-haplogroup Ages and Geographic links. Table S3. Haplogroup M geographic ranges and ages in kiloyears (kya). Table S4. Populations used in the AMOVA and k-means clustering analyses. Table S5. Mitochondrial DNA M haplogroup ages and coordinates for their respective geographic centers used in the correlation analysis. Table S6. Modern human oldest fossil dating in different regions of Asia and oldest archaeological dating at the eastern side of the Wallace Line. (XLSX 91 kb)
